# GIS-based non-grain cultivated land susceptibility prediction using data mining methods

**DOI:** 10.1038/s41598-024-55002-y

**Published:** 2024-02-23

**Authors:** Qili Hao, Tingyu Zhang, Xiaohui Cheng, Peng He, Xiankui Zhu, Yao Chen

**Affiliations:** 1 Shangluo Branch, Shaanxi Provincial Land Engineering Construction Group, Xi’an, 710075 China; 2 Shangluo Tea Research Institute, Shangluo, 726300 China; 3 Shangnan County Tea Industry Development Center, Shangluo, 726300 China

**Keywords:** Metaheuristic algorithms, Particle swarm optimization, Optimized extreme gradient boosting, Environmental management, Ecology, Natural hazards

## Abstract

The purpose of the present study is to predict and draw up non-grain cultivated land (NCL) susceptibility map based on optimized Extreme Gradient Boosting (XGBoost) model using the Particle Swarm Optimization (PSO) metaheuristic algorithm. In order to, a total of 184 NCL areas were identified based on historical records, and a total of 16 NCL susceptibility conditioning factors (NCLSCFs) were considered, based on both a systematic literature survey and local environmental conditions. The results showed that the XGBoost model optimized by PSO performed well in comparison to other machine learning algorithms; the values of sensitivity, specificity, PPV, NPV, and AUC are 0.93, 0.89, 0.88, 0.93, and 0.96, respectively. Slope, rainfall, fault density, distance from fault and drainage density are most important variables. According to the results of this study, the use of meta-innovative algorithms such as PSO can greatly enhance the ability of machine learning models.

## Introduction

Arable land serves as a crucial foundation for safeguarding food production, while also acting as the fundamental resource and spatial carrier to ensure food security, promote social development, and maintain ecological safety^1^. For generations, to prioritize the limited arable land resources for food production, steadfastly uphold the redline of arable land and the foundation of food security, and ensure that the quantity of arable land does not decrease, its quality improves, and its layout becomes more optimized, the Chinese government has been devoted to protecting arable land and ensuring the area dedicated to food cultivation by enacting stringent agricultural land protection measures^2^. The continuous optimization of China's agricultural industry structure and the rational regional layout have led to successive abundant harvests in food production, effectively ensuring the nation's food security. However, in recent years, driven by factors such as economic interests, some regions have simplistically understood agricultural restructuring as merely reducing food production. Consequently, phenomena such as unauthorized tree planting and pond digging in essential farmland, land transfer for non-food crop cultivation, and issues related to land occupation, marginalization, and fallow land have become increasingly prevalent. These factors have intensified the trend of "non-grain cultivation" in arable land, resulting in a considerable amount of non-grain cultivated land (NCL)^3^.

NCL refers to the agricultural practice of growing non-food crops on arable land primarily intended for cultivating grain crops^4^. This encompasses not only the cultivation of economically more profitable crops, fruits, or livestock but also includes fallow land and tree planting. NCL can lead to a series of ecological and environmental issues, such as declining soil fertility, increased greenhouse gas emissions, water scarcity, and pollution. Moreover, it poses a threat to the productive capacity of arable land and hinders the sustainable development of agricultural production^5^. Therefore, conducting research on NCL is of utmost necessity.

Since the earliest appearance of research on NCL in 2008, scholars have conducted investigations from different perspectives^6^. In terms of research content, the relevant studies on NCL mainly focus on four aspects: current characteristics, driving factors, impact effects, and policy measures^7,8^. Specific topics include the costs and benefits of cultivating arable land, the scale of agricultural land transfer, and government management systems^9^. As for the research field, early studies were concentrated in social sciences such as management, sociology, and economics, and later expanded to include geosciences like remote sensing monitoring, land management, and spatial planning^10^. In regard to research methods, there are primarily two categories for investigating NCL: one is statistical methods^11^, including multiple linear regression^12^, logistic regression^13^, Tobit model^14,15^, Probit model^16^, and random forest model^17^, while the other is geographic spatial analysis methods, encompassing spatial error model, spatial autocorrelation analysis, geographic weighted regression analysis, and spatial adjacency analysis^18^. Regarding the scale of research, many studies estimate the extent of NCL over large areas by comparing the ratios of grain and other crops, with grain-producing areas being the focus of research. However, there are fewer studies on areas with high NCL rates, particularly in hilly regions, and most studies are concentrated on macro-regional statistical analysis. Additionally, existing research has mostly relied on official statistical data and field surveys to estimate the extent of NCL in different regions^19^. There is still a lack of research that utilizes a small number of known NCL samples and combines natural factors to predict the spatial development range of NCL.

In recent years, the area of NCL in China has exhibited a significant increasing trend, with NCL occupying more than 27% of total arable land, and various regions showing distinct characteristics and distribution of NCL. As for Shaanxi Province, its southern region lies along the southern foot of the Qinling Mountains, belonging to a hilly and mountainous area with abundant rainfall. The severe occurrence of NCL, driven by natural factors, has led to a reduction in agricultural land, necessitating a quantitative assessment of the potential development range of NCL. This assessment will provide assurance for regional agricultural control.

To address the issue of controlling and managing NCL, the first step is to identify NCL, followed by predicting its spatial extent. This serves as the foundation for subsequent research on NCL. Accordingly, this study takes Chenggu County, Hanzhong City, Shaanxi Province, as the research area. Based on the natural driving factors of NCL and utilizing various data mining methods, we quantitatively predict the spatial development range of NCL within the county. This research aims to provide technical support and references for controlling the phenomenon of "non-grain cultivation" in hilly regions of China and promoting sustainable agricultural development.

The uniqueness of this research lies in its pioneering introduction of the concept of spatial prediction of NCL conversion. Furthermore, by taking counties as evaluation units and based on existing samples of NCL, this study quantitatively forecasts the spatial changes in NCL. Lastly, the methodological model employed in this research is also applied for the first time in the study of NCL prediction.

## Sample description of area

**Figure 1:**
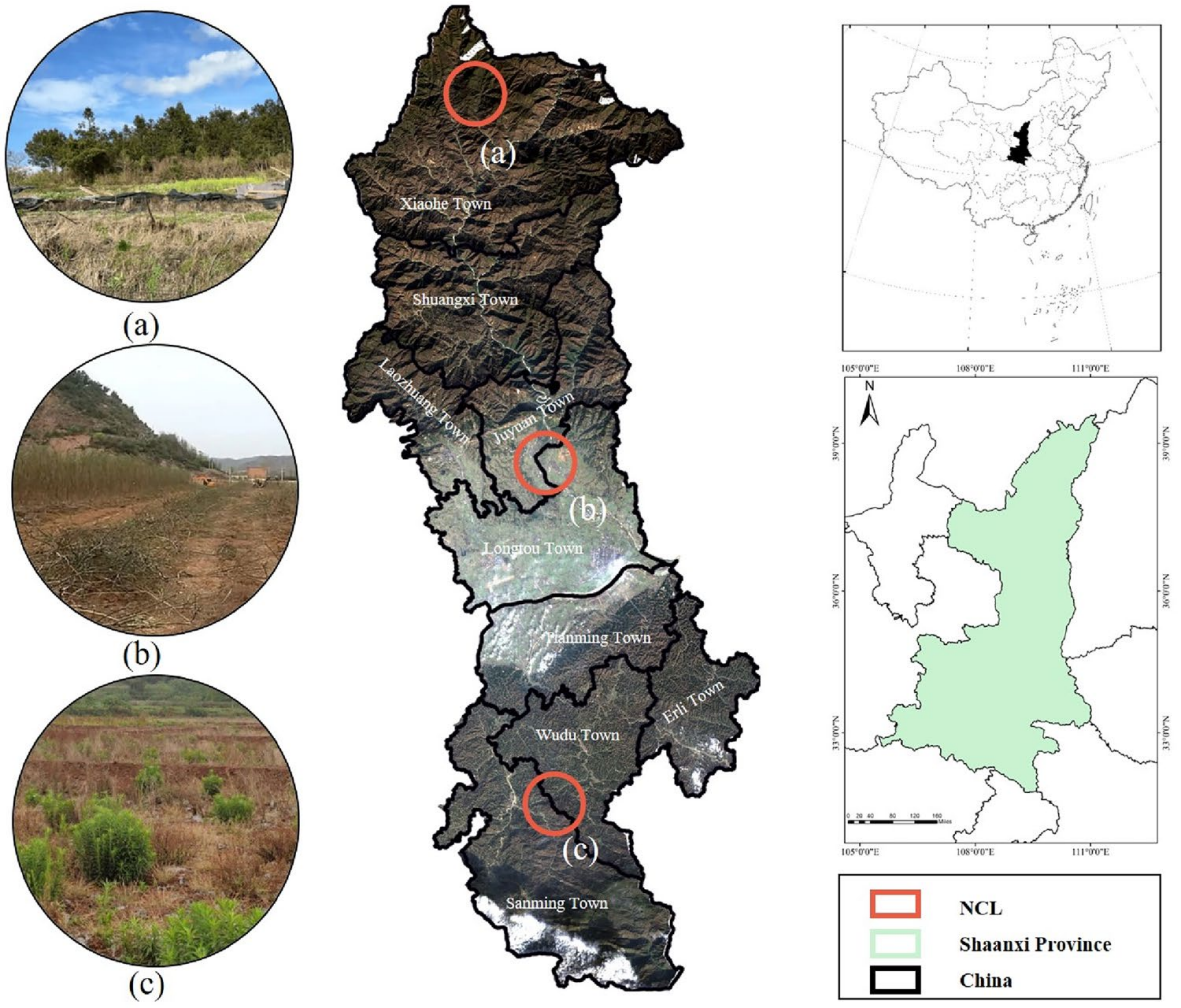
Study area location and areas with different NCL characteristics.

Chenggu County lies on the western side of Hanzhong City, Shaanxi Province, China. Its geographic coordinates range from 107°03′15″ to 107°30′45″ east longitude and from 32°45′15″ to 33°40′50″ north latitude (Fig. 1). The county covers an area of 2265 square kilometers. The terrain of the research area stretches in the north–south direction, with narrowness in the east–west direction. The elevation is higher in the north and south, and lower in the central part, sloping from south to north. The average elevation is 806.6 m, with the highest peak reaching 3289 m and the lowest point at 895 m. The relative elevation difference is as significant as 2135.6 m. The area is composed of mountains, plains, and hilly landscapes, covering 79.46%, 13.78%, and 6.76% of the total area, respectively^20^.

The climate in the research area belongs to the northern subtropical monsoon climate. The average annual temperature is 14.2 °C, and the average annual rainfall is 412 mm. The runoff is mainly generated by precipitation and consists of surface water and groundwater, with surface water being the dominant component. During the year, the high-water period (May to August) accounts for 55.1% and 46.3% of the total annual runoff, with a dry month embedded in it (June)^21^.

The vegetation type in the research area belongs to the northern subtropical zone, characterized by a belt of mixed coniferous and deciduous broad-leaved forests. Due to significant differences in landforms, altitude, and elevation, the vertical distribution of forests exhibits marked variations and follows a regular pattern.

In terms of soil resources, the county has seven soil types and sixteen subtypes, with predominant ones being yellow–brown soil, paddy soil, and yellow-cinnamon soil. According to the ‘Regional Geology of Shaanxi Province’, as of the end of 2022^22^, the total area of cultivated land in the research area is 23,800 hectares (including 18,733 hectares of paddy fields, 4932 hectares of dry fields, and 120 hectares of irrigated fields), with 1,706 hectares of temporary cultivated land and 36,667 hectares of perennial grain and oil crops. Based on the statistics released by the local government, the total rural labor force population reached 233,000, among which 139,400 people were engaged in migrant work, accounting for 59.8% of the total population^23^. The massive migration of rural labor has led to an extreme ‘hollowing out’ phenomenon in the age structure of the rural permanent population, resulting in a shortage of labor for cultivation of the land. Therefore, the situation of 'non-grain cultivation' on the land is becoming increasingly severe, thus requiring an urgent prediction of the spatial extent of non-grain cultivated land, which can guide the development planning and land protection implementation in the research area.

## Methodologies

### Research flow

**Figure 2:**
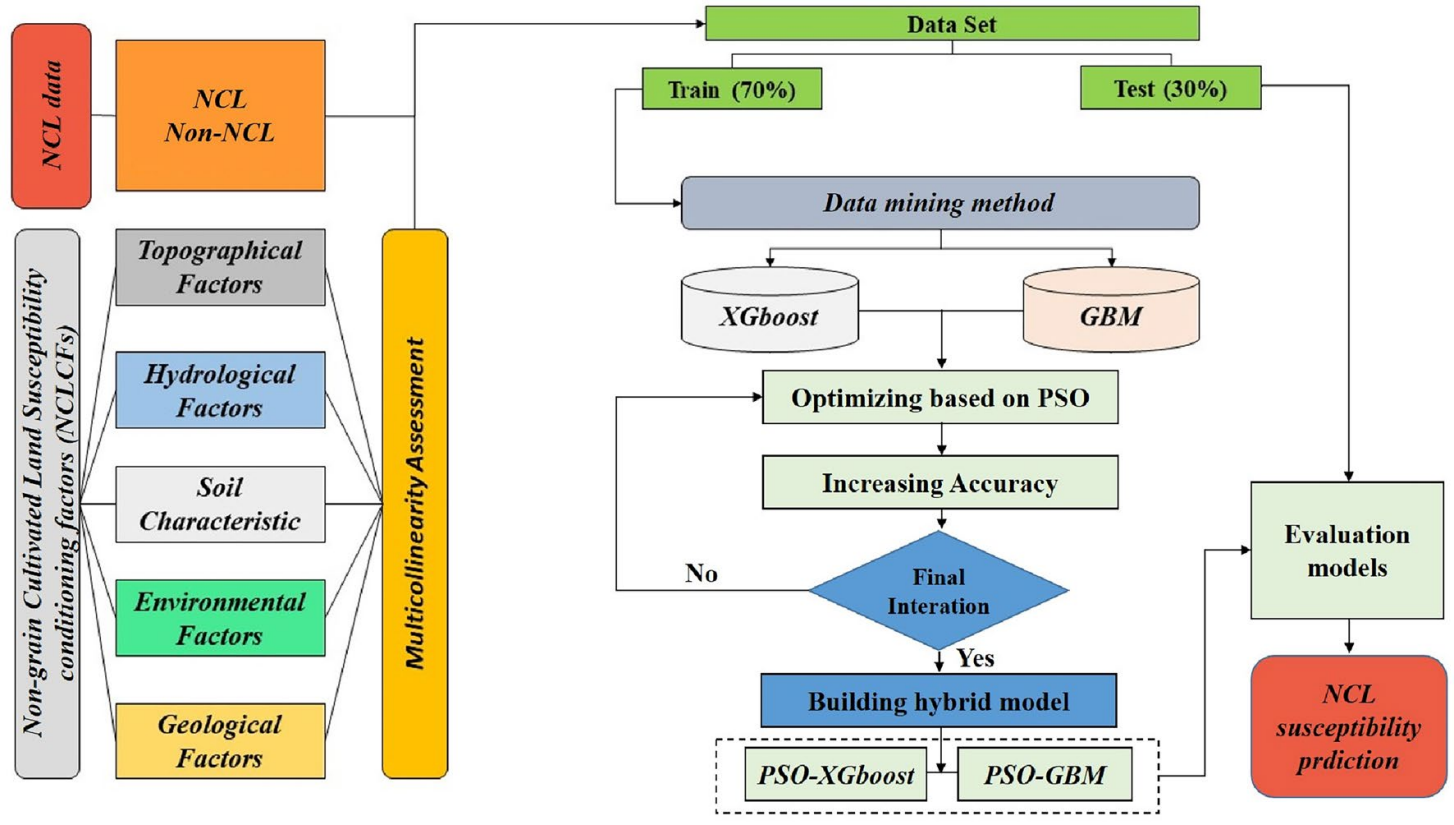
Research flow.

The NCL susceptibility prediction study includes four main parts: (1) screening and analysis of the influencing factors of NCL; (2) construction of the NCL susceptibility prediction model; (3) NCL susceptibility prediction; and (4) evaluation of the prediction results. The Research flow is shown in Fig. 2.

### Data sources

#### Non-grain cultivated land inventory

**Figure 3:**
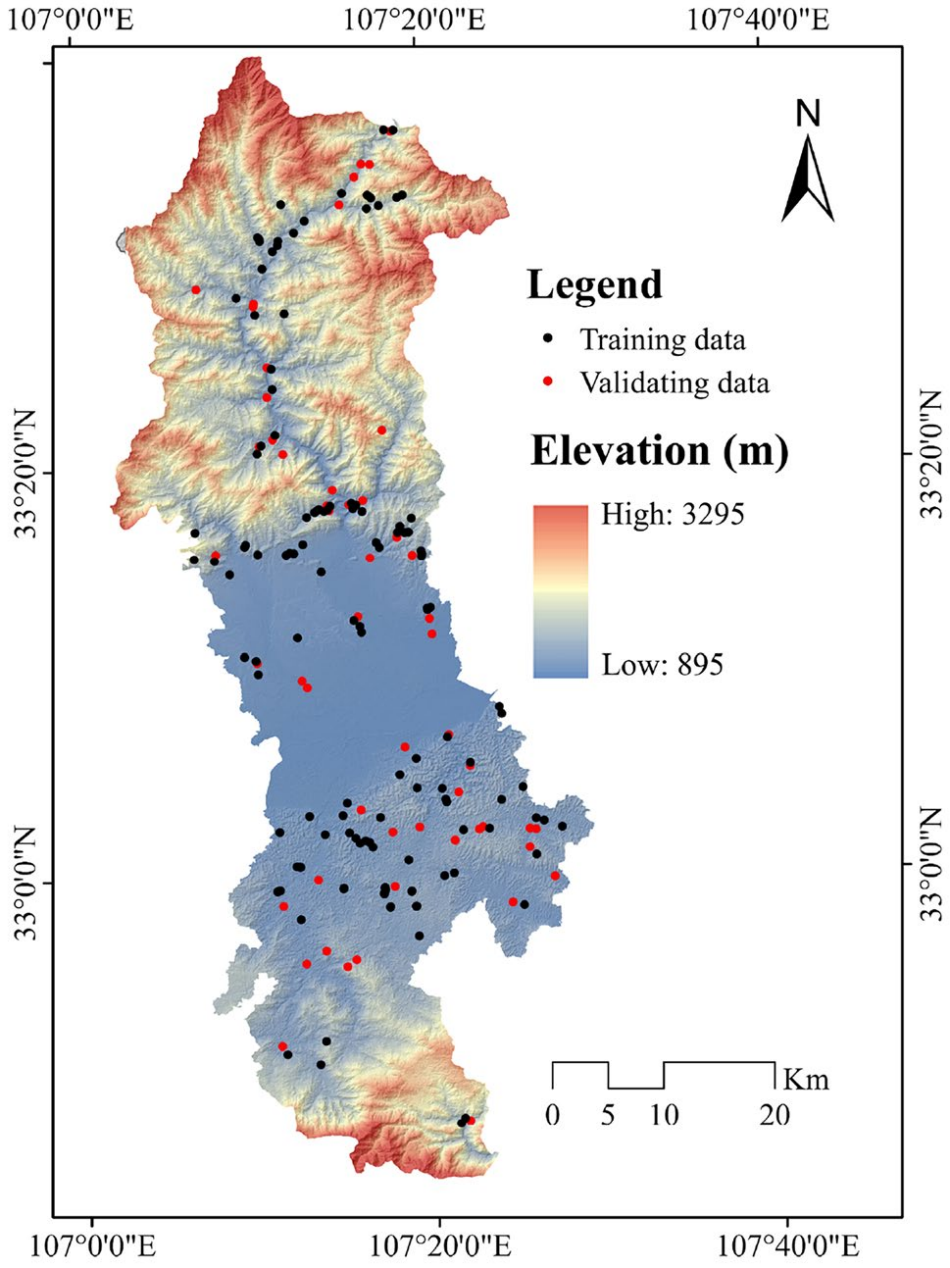
NCL inventory map.

The NCL locations were obtained based on information of Google Earth interpretation, field survey, and data released by local government, which derived in a total of 184 NCL locations. For determining the non-NCL locations, GIS software was applied, and 184 locations were randomly selected. In order to decreasing the bias of modeling, we generated non-NCL points by 200 m distance for NCL. At each point, the data was divided into training samples and testing samples in a ratio of 7/3, thus forming the training dataset and the testing dataset together (Fig. 3).

#### Analysis of non-grain cultivated land susceptibility conditioning factors (NCLCFs)

**Figure 4:**
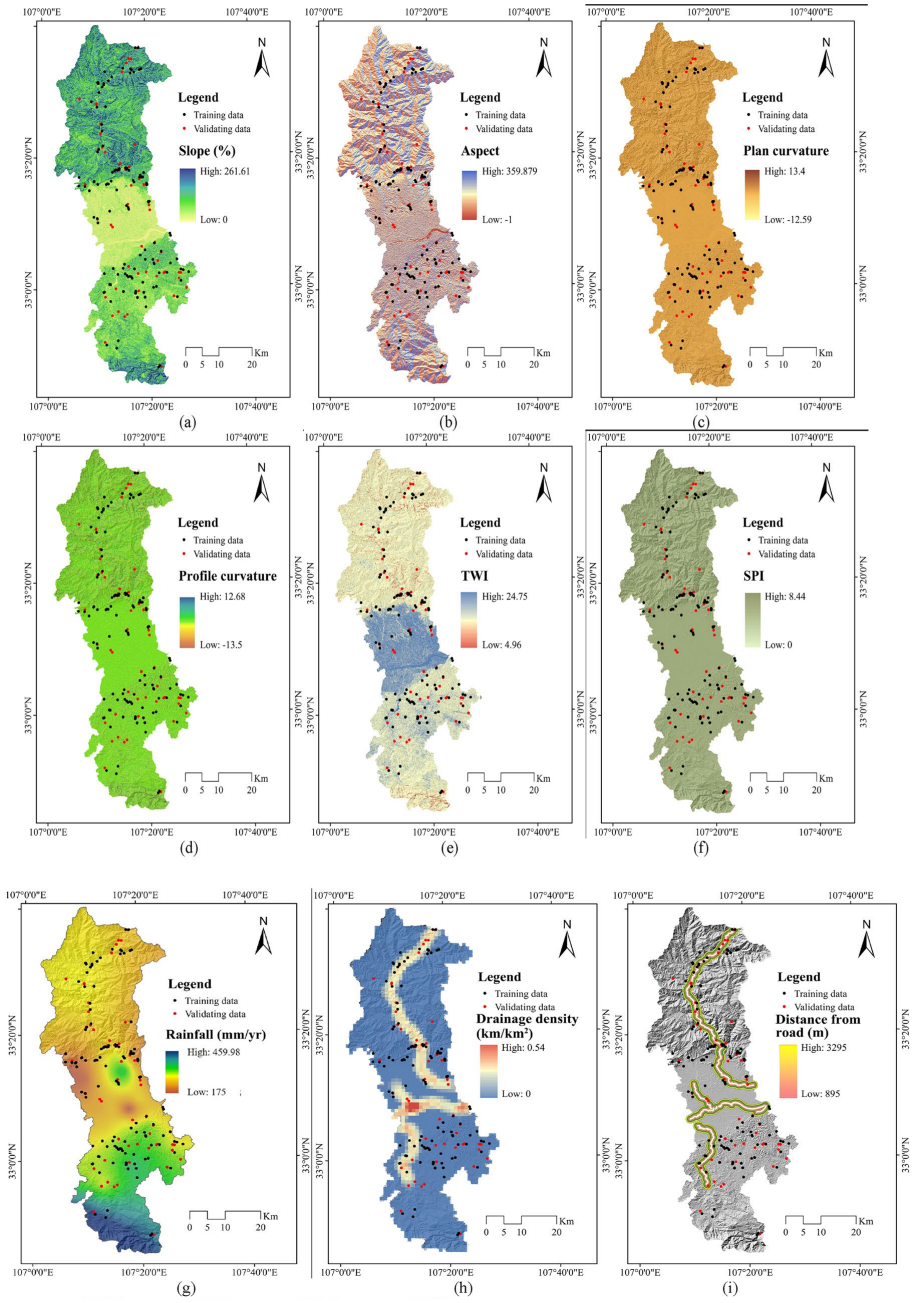

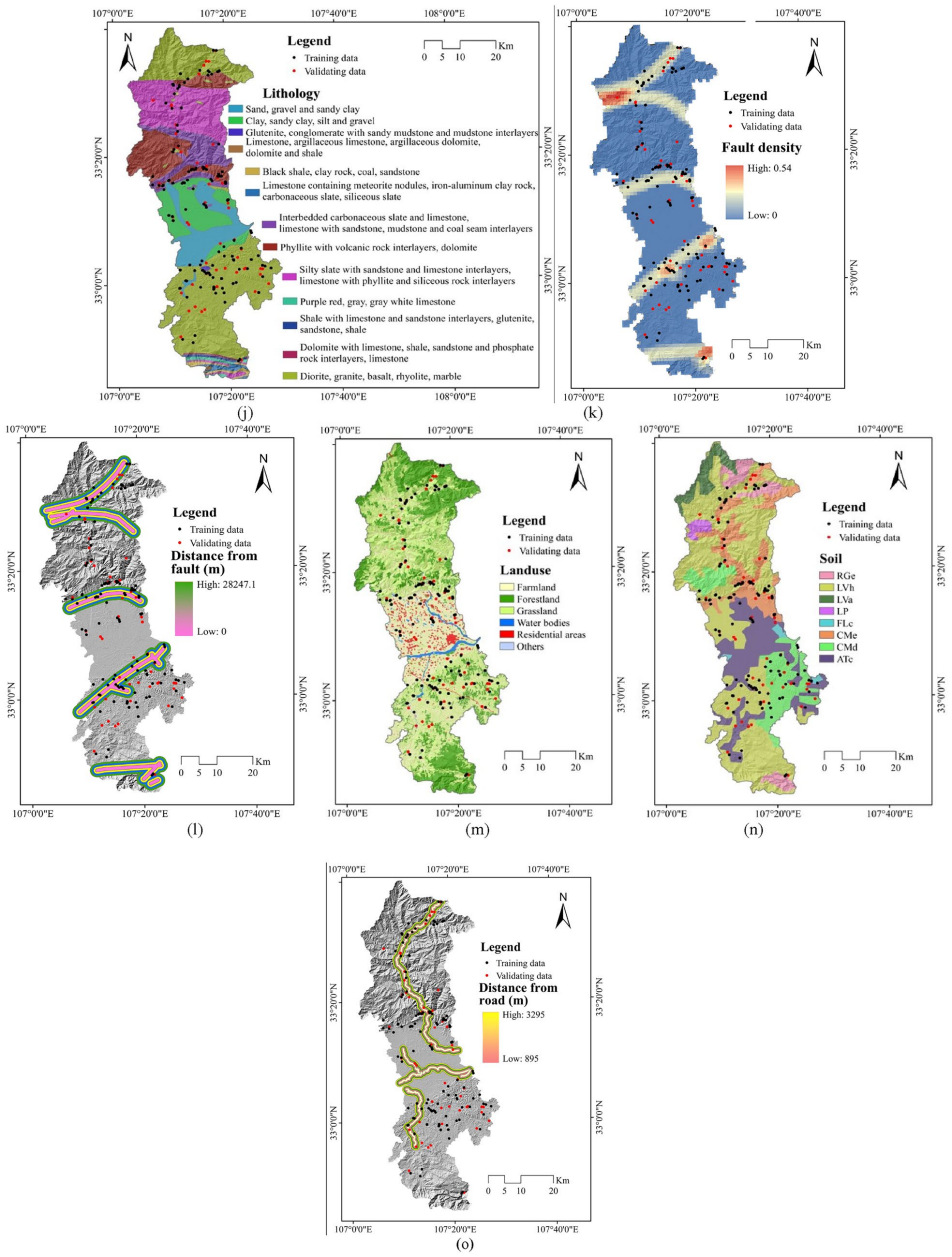
Typical NCL factors map: (**a**) Slope; (**b**) Aspect; (**c**) Plan curvature; (**d**) Profile curvature; (**e**) TWI; (f) SPI; (**g**) Rainfall; (h) Drainage density; (**i**) Distance from river; (**j**) Lithology; (**k**) Fault density; (**l**) Distance from fault; (**m**) Landuse; (**n**) Soil; (**o**) Distance from road.

Currently, there is no unified consensus on the factors influencing NCL. Therefore, based on historical research materials and on-site field investigations^24–28^, 16 appropriate Non-grain Cultivated Land Susceptibility conditioning factors (NCLSCFs) were chosen for modelling NCL susceptibility in accordance with topographical, geological, hydrological, climatological and environmental situations. Alongside this, a systematic literature review has also been performed on NCL modelling to aid in the identification of the most suitable NCLSCFs for this study. The NCLSCF maps were shown in Fig. 4.

(1) Topographical factors

The occurrences of NCL and their recurrent frequency are very much dependent on topographical factors of an area. Several topographical factors like slope, elevation, curvature, etc. are triggering parameters for the development of NCL activities^29^. Here, six topographical factors were chosen: altitude, slope, aspect, plan and profile curvature and topographic wetness index (TWI). All these factors also perform a considerable part in NCL development in study area. These factors were prepared using shuttle radar topographical mission (SRTM) sensor digital elevation model (DEM) data with 30 m resolution in the ArcGIS software. The output topographical factors of altitude ranges from 895 to 3289 m (Fig. 3), slope map 0–261.61%, aspect map has nine directions (flat, north, northeast, east, southeast, south, southwest, west, northwest), plan curvature − 12.59 to 13.40, profile curvature − 13.05 to 12.68 and TWI 4.96 to 24.75. The following equation was applied to compute TWI:

1 $$TWI = Ln\frac{\propto }{\mathrm{tan\beta }+\mathrm{ C}}$$

where, ∝ specifies flow accumulation, *β* specifies slope and C is the constant value (0.01).

(2) Hydrological factors

Sub-surface hydrology is treated as the activating mechanism for the happening of NCL, as water performs a significant part in the soil moisture content. Therefore, four hydrological factors, namely drainage density, distance from river, stream power index (SPI) and annual rainfall, for modelling NCL susceptibility were chosen^30^. Here, SRTM DEM data of 30 m spatial resolution was used to map the first three hydrological variables. Drainage density and distance from river map was prepared using line density extension and Euclidean extension tool respectively in GIS platform. The following formula was applied to compute SPI.

2 $$SPI = As*\tan \beta$$

where, *As* specifies the definite catchment area in square meters and β specifies the slope angle in degrees. The precipitation map of the area was derived from the statistics of 19 climatological stations around the province with a statistical period of 25 years and in accordance with the kriging interpolation method in GIS platform. The output drainage density value ranges from 0 to 1.68 km/km^2^. Meanwhile, the value of distance from river ranges between 0 and 9153.93 m, average annual rainfall varies from 175 to 459.98 mm and the value of SPI ranges from 0 to 8.44.

(3) Geological factors

The characteristics of rock mass, i.e., lithological characteristics of an area, significantly impact on NCL activities^31^. Therefore, in NCL susceptibility studies geological factors are indeed commonly used as input parameters to optimize NCL prediction assessment. In the current study, three geological factors (namely lithology, fault density and distance from fault) were chosen. The lithological map and fault lines were obtained in accordance with the geological map of study gathered from local government at a scale of 1:100,000. Fault density and distance from fault factor map was prepared using line density extension and extension tool respectively in GIS platform. In this area, the value of fault density varies from 0 to 0.54 km/km^2^ and distance from fault ranges from 0 to 28,247.1 m respectively. The lithological map in this area is presented in Fig. 4b.

(4) Environmental factors

Several environmental factors can also be significant triggering factors for NCL occurrence in mountainous or hilly regions^32^. Here, land use land cover (LULC), soil and distance from road were selected as environmental variables for predicting of NCL susceptibility. The LULC map was obtained in accordance with Landsat OLI 8 satellite images applying the maximum probability algorithm in the ENVI. Soil texture map was prepared based on the soil map of study area. The road map of this area was digitized from the topographical map by the local government. The output LULC factor was classified into six land use classes, while the soil map was classified into eight soil texture groups and the value of distance from road ranges from 0 to 31,248.1 m.

### Evaluation method of NCLCFs

As the NCLSCFs are selected artificially and their dimensions, as well as the quantification methods of data, are derived through mathematical operations, as subsequent input data for modeling, there may be potential multicollinearity problems among the NCLSCFs^33^. Such problems arise due to precise or highly correlated relationships between NCLSCFs, which can lead to model distortion or difficulty in estimation. In light of this, to avoid potential multicollinearity problems, this study examines the variance inflation factor and tolerance index to assess whether there exists multicollinearity among the NCLSCFs.

The MC analysis was conducted among the chosen NCLSCFs to optimize the NCL susceptibility model and its predictions^34^. TOL and VIF statistical tool were used to test MC using SPSS software. Studies indicate that there is a multicollinearity issue if VIF value is > 5 and TOL value is < 0.10. TOL and VIF were measured applying the following formula:

3 $$TOL=1-{R}_{j}^{2}$$

4 $$VIF=\frac{1}{TOL}$$

where, *R*^2^ represents a regression value of j on other various factors.

### Mechanism of NCL susceptibility model

This section details the machine learning models of GBM and XGB, as used in NCL susceptibility studies.

#### Gradient boosting model (GBM)

In prediction performance analysis, GBM is one of the most popular machine learning methods, more frequently applied by researchers in different fields and treated as a supervised classification technique. A variety of classification and regression issues are also often solved by the GBM method, which was first proposed by Friedman^35^. This model is based on the ensemble of different weak prediction models such as decision trees, and is therefore considered as one of the most important prediction models. Three components are required in GBM model, namely a loss operate, a weak learner prediction, and an optimization of the loss function in which an additive function is necessary to include weak learners within the model. In addition to the above mentioned components, three important tuning parameters (namely n-tree, tree depth and shrinkage, i.e., the maximum number of trees, highest possible interaction among the independent variables and the learning rate respectively) is also required to build a GBM model^36^. The advantage of such a model is that it has capacity to determine the loss function and weak learners in a precise way. It is complex to obtain the solution of optimal estimation applying the loss function of (*y*, *f*) and weak learner of ℎ(*x*, θ). Thus, to solve this problem, a new operate ℎ(*x*, θ*t*) was planned to negative gradient {*gt*(*xi*)}*i* = 1 along with the observed data:

5 $${g}_{t}(x) ={{E}_{y} [\frac{a\psi (y,f(x))}{af(x)}|x]}_{f(x)={f}^{t-1}(x)}$$

This new operate is greatly associated with − (*x*). This algorithm can permit us to develop aleast square minimization from the method by applying the following equation:

6 $$(\mathrm{\rho t},\mathrm{ \theta t})=\mathrm{arg min}\sum_{i=1}^{N}{[-{\text{gt}}(\mathrm{xi }) +\mathrm{ \rho h}(\mathrm{xi },\uptheta ]}^{2}$$

#### Extreme gradient boosting (XGB)

Chen & Guestrin then went on to introduce the XGB algorithm. It indicates the advance machine learning method, and is more efficient than the others^37^. The algorithm of XGB is based on classification trees and the gradient boosting structure. Gradient boosting framework is used in an XGB model by the function of parallel tree boosting. This algorithm is chiefly applied for boosting the operation of different classification trees. A classification tree is usually made up of various regulations to classify each input factor as the function of prejudice variables in a plot construction. This plot is developed as a individual tree and leaves are appointed with respective scores, which convey and choose the respective factor class, i.e., categorical or ordinal. The loss function is used in the XGB algorithm to train the ensemble model; this is known as regularization, which deals specifically with the severity of complexity trees^38^. Therefore, this regularization method can significantly enhance the performance of prediction analysis through alleviating any over-fitting problems. The boosting method, with the combination of weak learners, is used in XGB algorithm to optimally predict the result. Three parameters (i.e., General, Task and Booster) are applied to separate XGB models. The weighted averages of several tree models are then combined to form the output result in XGB. The following optimization function was applied to form the XGBoost model:

7 $$OF(\theta ) =\sum_{i=1}^{n}l\left({{\text{y}}}_{i}, {\overline{y} }_{i}\right)+\sum_{k=1}^{k}\upomega ({f}_{K})$$

where, $\sum_{i=1}^{n}l\left({{\text{y}}}_{i}, {\overline{y} }_{i}\right)$ is the optimization loss function of training dataset, $\sum_{k=1}^{k}\upomega ({f}_{K})$ is the regularization of the over-fitting phenomenon, *K* indicates the number of individual trees, *fk* is the ensemble of trees, and ${\overline{y} }_{i}$ and ${{\text{y}}}_{i}$ indicates the actual and predicted output variables respectively.

#### Particle swarm optimization (PSO)

Kennedy, an American social psychologist, developed the PSO algorithm based on the vector depending of seeking food by birds and their eating behavior^39^. It is a meta-heuristic-based simulation of a social model, often applied in behavioral studies of fish schooling, birds and swarming theory. The non-linear problems in our day-to-day research study will be solved by applying this PSO method. The PSO algorithm has been widely applied to determine the greatest achievable direction or direction to collect food, specifically for bird and fish intelligence. Here, birds are treated as particles, and they always search for an optimal result to the issue. In this model, bird is considered an individual, and the swarm is treated as a group like other evolutionary algorithms. The particles always try to locate the best possible solution for a respective problem using *n*-dimensional space, where *n* indicates the respective problem’s several parameters^40^. PSO consists of two fundamental principles: position and speed. This is the basic principle for the movement of each particle.

Hence, *x*^*t*^ = (*x*^*t*^, *x*^*t*^,…, *x*^*t*^) *and v*^*t*^ = (*v*^*t*^, *v*^*t*^, … , *v*^*t*^) is the position and speed for the changing particle position which is designed for ith particle in tth iteration. The given formula are used for the ith particle position and speed in (t + 1)th iteration.

Where, *x*^*t*^ is the previous *ith* position; *p*^*t*^ is the most excellent position; *g*^*t*^ is the best position; *r*_1_ and *r*_2_ indicates the random numbers within 0 and 1; ω is weights of inertia; *c*_1_ is coefficient and *c*_2_ is the social coefficient. Several type of methods are presented to weight the assignment of respective particles. Among them, standard 2011 PSO is the most popular and has been widely used among previous researchers. Here, standard 2011 PSO was used to calculate particle’s weight assignment using the following formula:

8 $$\omega =\frac{1}{2ln2}and {c}_{1}={c}_{2}=0.5+ln2$$

### Evaluation method of NCL susceptibility prediction

Evaluation is an important action to quantify the accuracy of each output method. In other words, the superiority of the output model is specified through a validation assessment^41^. Studies indicate that several statistical techniques can be applied to evaluate the accuracy of the algorithms; among them, the most frequently used technique is receiver operating characteristics-area under curve (ROC-AUC). Here, statistical techniques of sensitivity (SST), specificity (SPF), positive predictive value (PPV), negative predictive value (NPV) and ROC- AUC were all applied to validate and assess the accuracy of the models. These statistical techniques were computed in accordance with the four indices, i.e., true positive (TP), true negative (TN), false positive (FP) and false negative (FN)^42^. In this, correctly and incorrectly identified NCL susceptibility zones are represented through TP and FP, and correctly and incorrectly identified non-NCL susceptibility zones are represented through TN and FN respectively. The ROC is mostly used as a standard process to evaluate the accuracy of the methods. It is based on even and non-even phenomena. The output result of these techniques is such that a higher value represents good performance by the model, and a lower value represents poor performance. Applied statistical techniques of this study were measured through the following formula:

9 $${\text{SST}}=\frac{{\text{TP}}}{\mathrm{TP }+\mathrm{ FN}}$$

10 $${\text{SPF}}=\frac{{\text{TN}}}{\mathrm{FP }+\mathrm{ TN}}$$

11 $${\text{PPV}}=\frac{{\text{TP}}}{\mathrm{FP }+\mathrm{ TP}}$$

12 $${\text{NPV}}=\frac{{\text{TP}}}{\mathrm{TP }+\mathrm{ FN}}$$

13 $$AUC=\frac{\mathrm{\Sigma TP }+\mathrm{ \Sigma TN}}{\mathrm{P }+\mathrm{ N}}$$

## Results

### Result of NCLCFs evaluation

#### Multicollinearity analysis

The multicollinearity of all selected variables has been carried out in accordance with VIF and TOL limit. In this research, the ranges of VIF and TOL are 1.03–2.72 and 0.37–0.97 respectively. All the factors in this analysis are free from multicollinearity and have thus been considered for NCL susceptibility assessment. The value of VIF and TOL of all chosen variables is indicated in Table 1.

**Table 1:** Multicollinearity analysis.

Variables	VIF	Tolerance
Aspect	1.03	0.97
Altitude	2.37	0.42
Drainage density	1.45	0.69
Distance from river	1.36	0.73
Fault density	2.72	0.37
Distance from fault	2.31	0.43
Landuse	1.31	0.76
Lithology	1.52	0.66
Plan curvature	1.51	0.66
Profile curvature	1.41	0.71
Distance from road	1.36	0.74
Rainfall	1.73	0.58
Slope	2.06	0.49
Soil	1.47	0.68
SPI	1.06	0.94
TWI	1.93	0.52

#### Tune and optimization parameters

**Figure 5:**
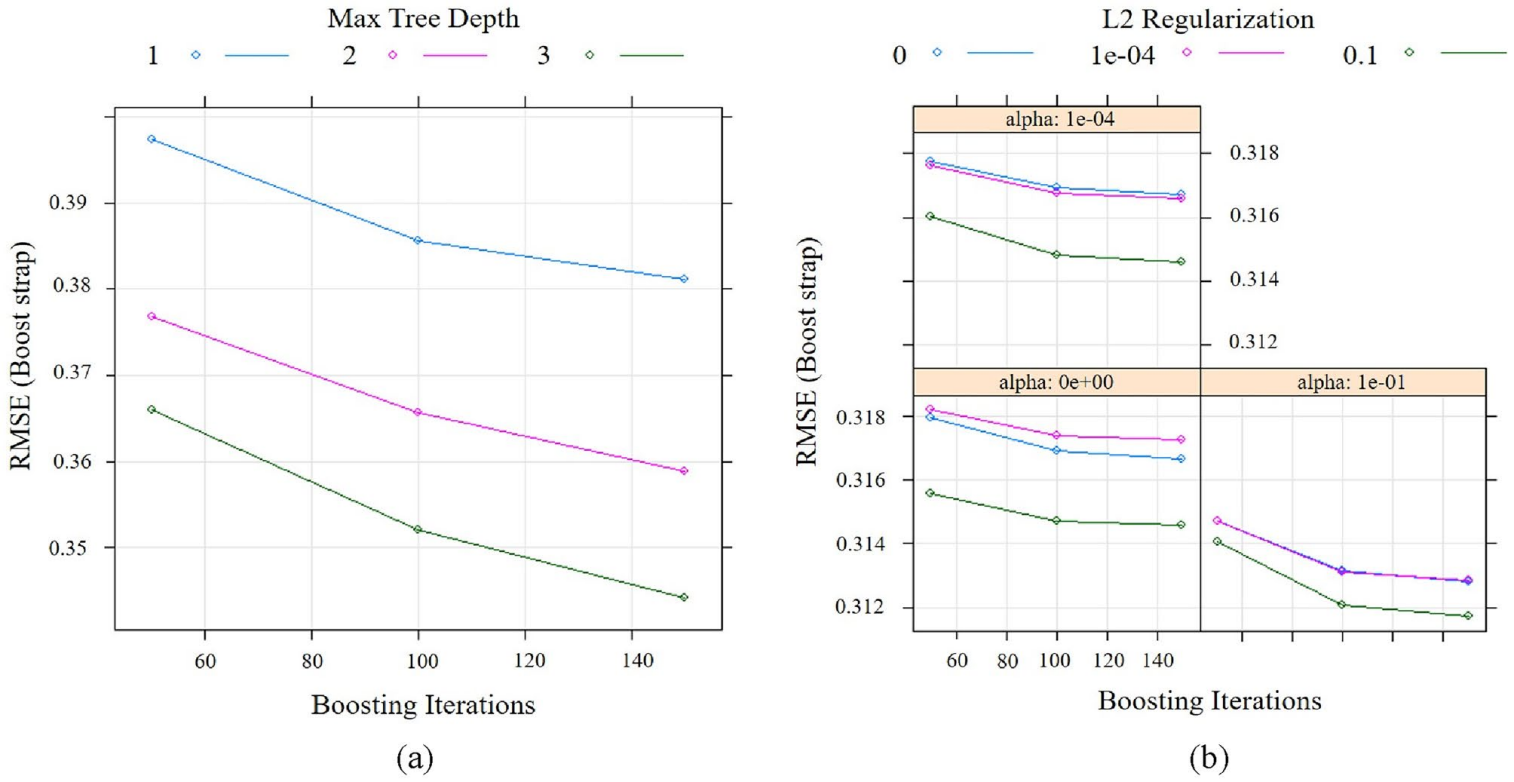
Tune parameters: (**a**) GBM and (**b**) XGB.

**Figure 6:**
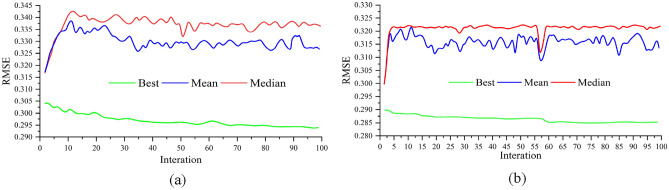
Result of optimization parameters based on PSO algorithm in XGB model and GBM model: (**a**) GBM and (**b**) XGB.

The hyper-parameter tuning, or tune parameter model, has been used to develop models for ML algorithms. We require that the system makes this observation in a true machine learning manner and instantly selects the proper training set. As a result, hyper-parameter configuration refers to simulations of tuning parameters in search of the best classification problem. In Fig. 5a, the tuning parameters of GBM are indicated by considering boosting iterations and RMSE based on Max Tree Depth. On the other hand, in the XGB model, the relationship between boosting iterations and its associated RMSE has been identified with the help of L2 regularization (Fig. 5b). The association between iteration and its related Error has been estimated, considering mean, median and best values in GBM model and XGB model in accordance with PSO algorithm (Fig. 6). The value of n-rounds, lamba, alpha, eta and Error for XGB are 150, 0.10, 0.10, 0.30 and 0.31 respectively. The value of n-rounds, lamba, alpha, eta, population size and Error in PSO-XGB are 557, 1.0, 3.0, 20 and 0.28 respectively (Table 2). The value of n-trees, max-features, min-samples-split, min-samples-leaf, tree-depth, learning rate in GBM are 300, 1, 3, 1, 4 and 0.10 respectively. The value of n-trees, max-features, min-samples-split, min-samples-leaf, tree-depth, learning rate in PSO-GBM are 437, 0.42, 2, 3, 6 and 0.28 respectively (Table 3).

**Table 2:** Result of determining the best parameters in XGB model based on PSO algorithm.

Parameters	XGB	PSO-XGB
n-rounds	150	557
Lamba	0.1	1
Alpha	0.1	− 0.11184
Eta	0.3	3
Population size	–	20
RMSE	0.31172	0.28542

**Table 3:** Result of determining the best parameters in GBM model based on PSO algorithm.

Parameters	GBM	PSO-GBM
n-trees	300	437
Max-features	1	0.427906
Min-samples-split	3	2
Min-samples-leaf	1	3
Tree depth	4	6
Learning rate	0.100000	0.287674

#### Result of evaluating accuracy

The validation of all predictive methods has been done considering different indices. The value of sensitivity, specificity, PPV, NPV and AUC in the GBM model with training datasets are 0.89, 0.85, 0.86, 0.89 and 0.95 respectively. On the other hand, using validation datasets, the value of sensitivity, specificity, PPV, NPV and AUC in the GBM model are 0.86, 0.81, 0.82, 0.86 and 0.92 respectively. The value of sensitivity, specificity, PPV, NPV and AUC in the PSO-GBM model with training datasets are 0.95, 0.92, 0.93, 0.98 and 0.97 respectively. Using validation datasets, the value of sensitivity, specificity, PPV, NPV and AUC in the GBM model are 0.92, 0.86, 0.85, 0.92 and 0.95 respectively.

**Figure 7:**
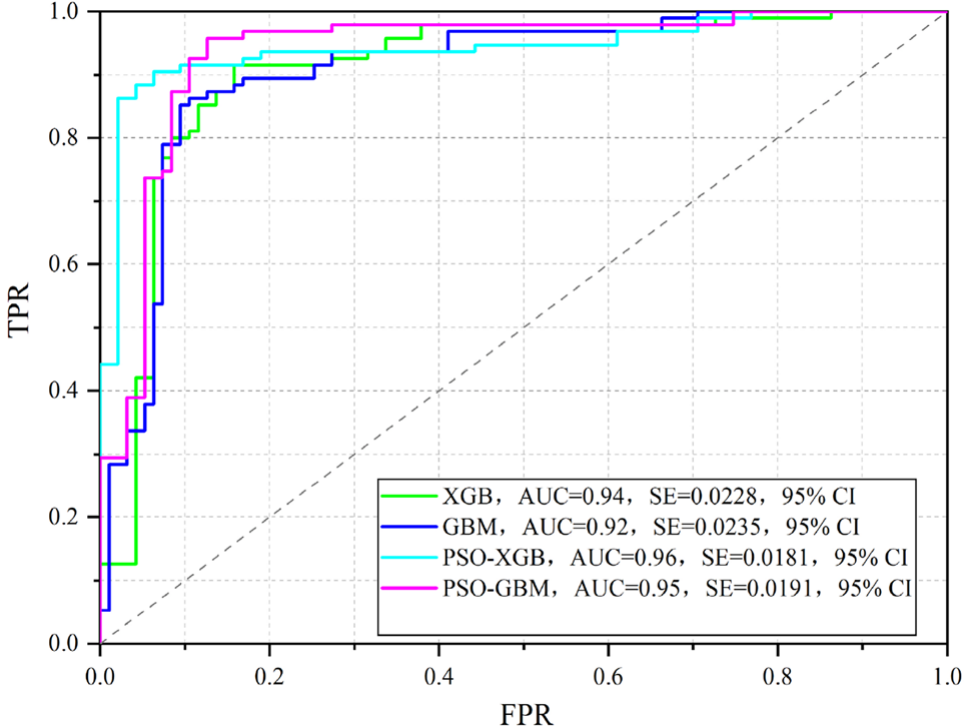
The ROC curve analysis for four NCL susceptibility prediction methods in validation stage.

The value of sensitivity, specificity, PPV, NPV and AUC in the XGB model with training datasets are 0.98, 0.94, 0.93, 0.97 and 0.98 respectively. On the other hand, with validation datasets, the value of sensitivity, specificity, PPV, NPV and AUC in the XGB model are 0.91, 0.88, 0.88, 0.91and 0.94 respectively (Table 4). With training datasets, the values of sensitivity, specificity, PPV, NPV, and AUC in the PSO-XGB model are 0.98, 0.95, 0.94, 0.99, and 0.99, respectively. In the PSO-XGB model, the values of sensitivity, specificity, PPV, NPV, and AUC are 0.93, 0.89, 0.88, 0.93, and 0.96, respectively, when validation datasets are taken into account (Fig. 7). Here PSO- XGB is the most optimal model, followed by XGB and GBM model, considering both training and validation datasets.

**Table 4:** The results of evaluating accuracy in training and test dataset.

Models	Stage	Parameter				
		Sensitivity	Specificity	PPV	NPV	AUC
GBM	Train	0.89	0.85	0.86	0.89	0.95
	Validation	0.86	0.81	0.82	0.86	0.92
XGB	Train	0.98	0.94	0.93	0.97	0.98
	Validation	0.91	0.88	0.88	0.91	0.94
PSO-GBM	Train	0.95	0.92	0.93	0.98	0.97
	Validation	0.92	0.86	0.85	0.92	0.95
PSO-XGB	Train	0.98	0.95	0.94	0.99	0.99
	Validation	0.93	0.89	0.88	0.93	0.96

### Result of NCL susceptibility prediction

**Figure 8:**
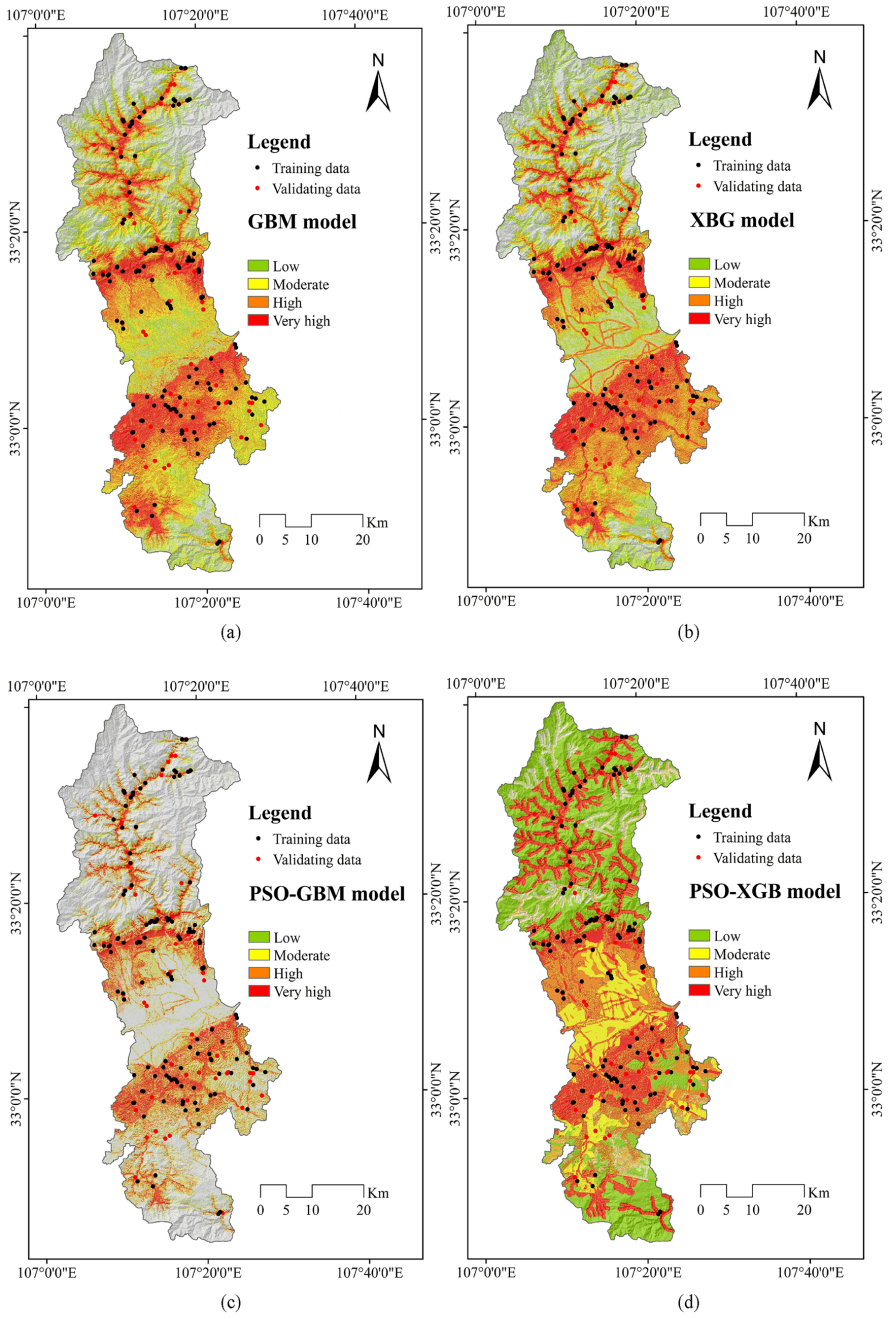
NCL susceptibility maps based on the three models: (**a**) GBM, (**b**) XGB, (**c**) PSO-GBM and PSO-XGB.

**Figure 9:**
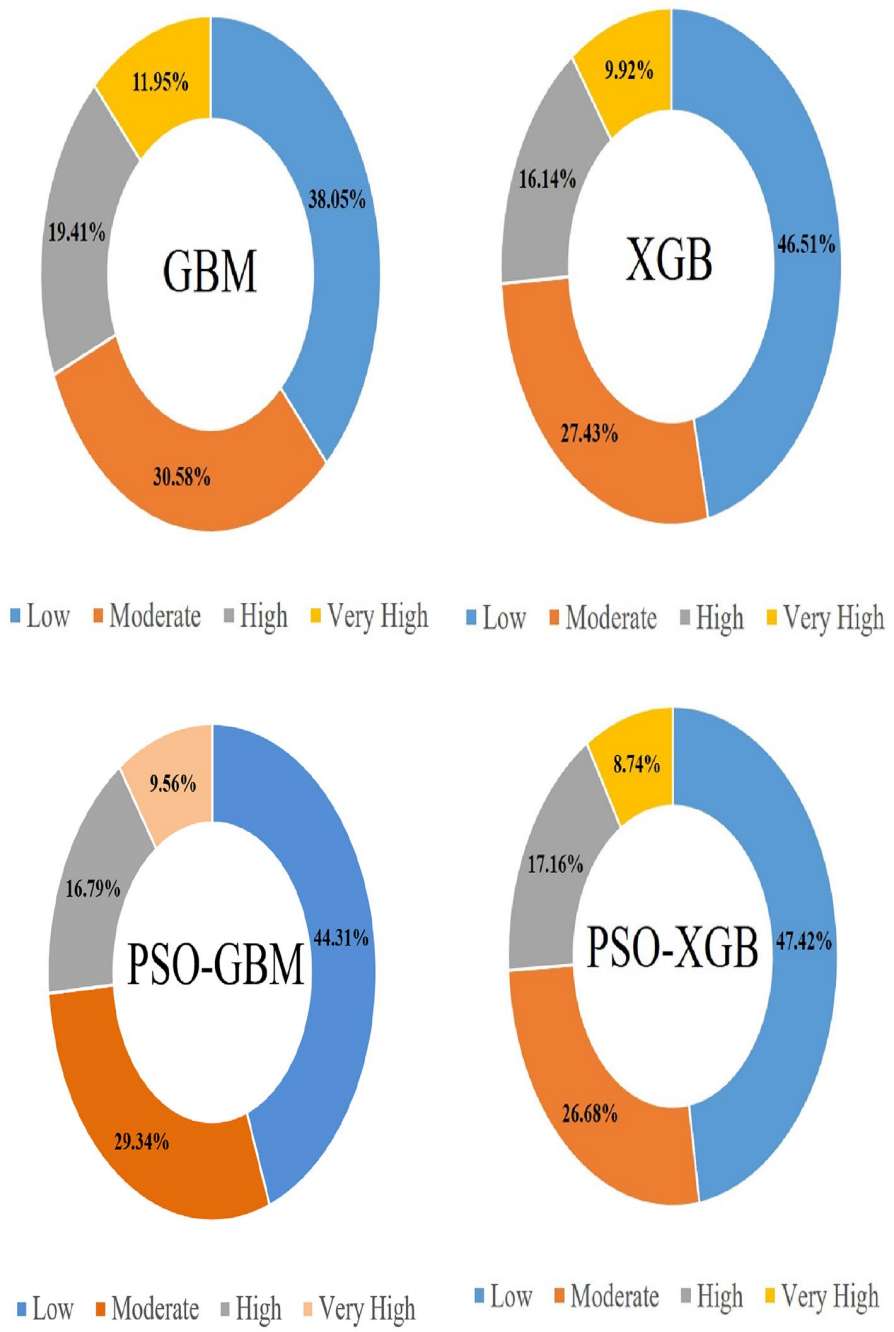
NCL susceptibility percent classes’ area.

**Figure 10:**
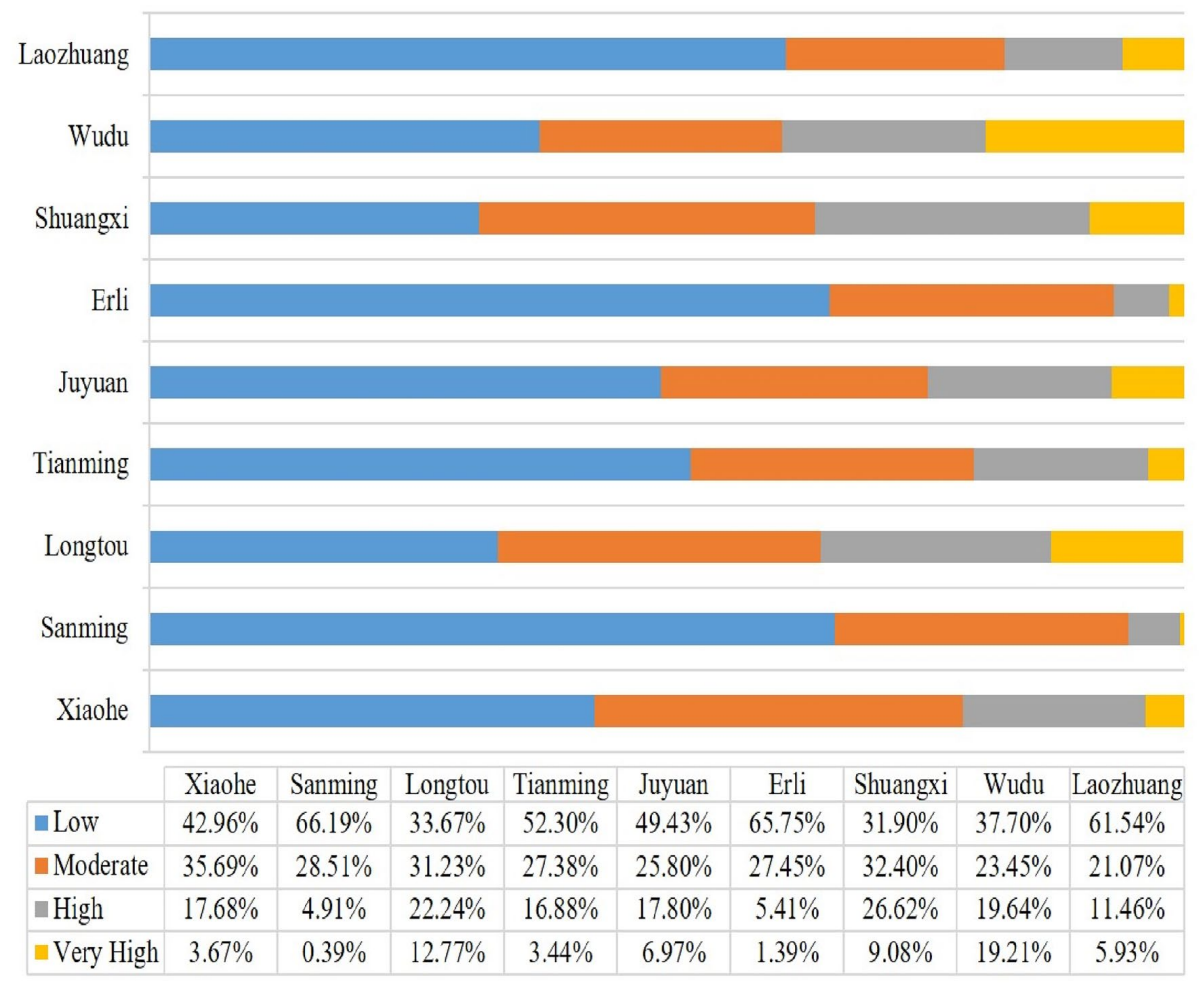
The percentage covered by NCL susceptibility area for 9 towns of Chenggu County based on the PSO- XGBoost model.

The Non-grain Cultivated Land Susceptibility prediction has been done with each of GBM, XGB and PSO-XGB models. The spatial coverage of very highly and highly NCL-susceptible areas is most importantly focused in the middle and southern portion of the case study (Fig. 8). The areal coverage for low, moderate, high and very high NCL susceptibility zones in the GBM algorithm is 861.83 km^2^, 692.64 km^2^, 439.64 km^2^ and 270.67 km^2^ respectively. The areal coverage for low, moderate, high and very high NCL susceptibility zones in the XGB model is 1053.45 km^2^, 621.29 km^2^, 365.57 km^2^ and 224.69 km^2^ respectively (Table 5). The areal coverage for low, moderate, high and very high NCL susceptibility zones in the PSO-GBM model is 1003.62 km^2^, 664.55 km^2^, 380.29 km^2^ and 216.53 km^2^ respectively. The areal coverage for low, moderate, high and very high NCL susceptibility zones in the PSO-XGB model is 1074.06 km^2^, 604.30 km^2^, 388.67 km^2^ and 197.96 km^2^ respectively (Fig. 9). The results of the percentage covered by NCL for 9 towns of Chengu County based on the PSO- XGBoost model are shown in Fig. 10. Based on these results, it is observed that Wudu, Longtou and Shuangxi Towns with 19.21%, 12.77% and 9.08%, respectively, have the highest percentage of NCL susceptibility area with very high sensitivity.

**Table 5:** NCL susceptibility classes’ area.

Algorithms	Area	Susceptibility class			
		Low	Moderate	High	Very high
GBM	km^2^	861.83	692.64	439.64	270.67
XGB	km^2^	1053.45	621.29	365.57	224.69
PSO-GBM	km^2^	1003.62	664.55	380.29	216.53
PSO-XGB	km^2^	1074.06	604.30	388.67	197.96

### Variable importance

**Figure 11:**
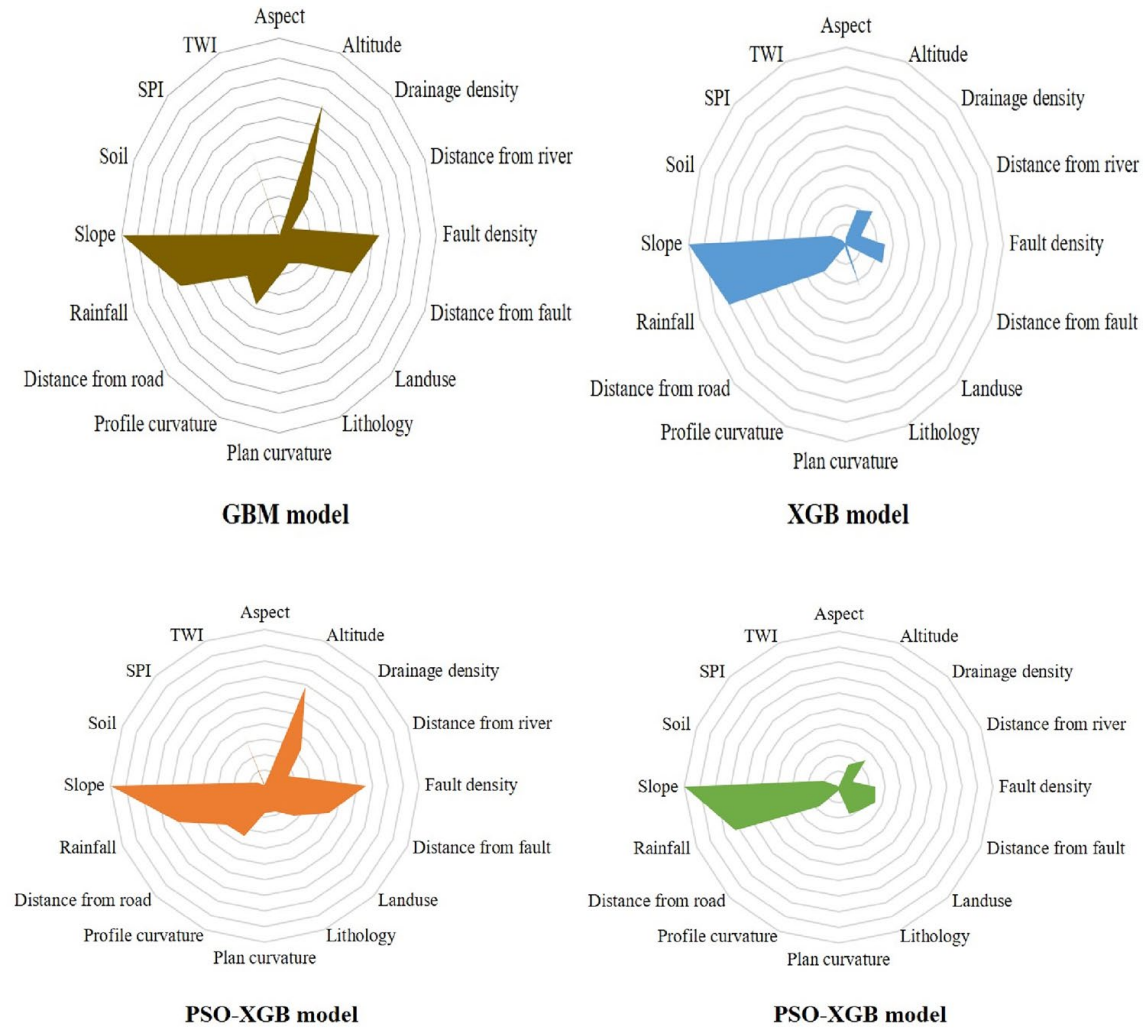
Result of importance value in three models.

In all the predicted models, the significance value of the 16 causative variables has been evaluated. Because the variables included in this study had a value of larger than 0, they were used to the NCL susceptibility model. In the GBM model, higher importance is associated with some parameters, i.e., slope, altitude, rainfall, fault density, distance from fault and TWI, etc. On the other hand, in the XGB model, slope, rainfall and fault density show the highest importance. For PSO-GBM model, slope, altitude, rainfall, fault density, distance from fault and TWI are the important variables. In the PSO-XGB model, the higher importance is associated with slope, rainfall, fault density, distance from fault and drainage density (Fig. 11).

## Discussion

NCL is a complicated process that occurs as a consequence of a mix of internal and external causal and triggering variables. It's crucial to understand these elements and assess their potential impact on NCL when assessing NCL susceptibility. A key premise that is followed in most NCL study approaches, especially quantitative ones, is that if the same combination of causative variables that caused previous NCL in one region is then repeated in other places, NCL may be predicted again. As a result, it is critical to analyze causative variables and assess their prospective link with previous NCL in the region, this will serve as the foundation for predicting potential NCL locations in the future.

This study uses a hybridization approach to estimate the NCL susceptibility in more accurate ways. In this perspective the GBM, XGB, PSO-GBM and PSO-XGB model has been considered for NCL susceptibility evaluation. The efficiency of the XGBoost method is higher than another method used in this investigation. Furthermore, the PSO-XGB hybrid model has a rapid convergence rate, minimal error, and good precision in the adaptive optimization problem. In many studies, XGBoost model has been used to model the susceptibility of NCL in different parts of the world. Most of these studies did not use the optimal parameters of the model and only entered the parameters into the model by default. The targeted optimization iteration curve of the hybrid model is presented, with the PSO- XGB hybrid model and PSO-GBM hybrid model showing a good effect as well. Moreover, it is evident from the results that the PSO method can effectively improve the performance of the model. It is worth thinking about whether the proposed PSO-XGBoost model may be used to estimate the HL of building systems, for instance. On the basis of the architecture presented, a combined process including the XGBoost model and the PSO algorithm was created. Prior to executing the XGBoost model optimization, the PSO algorithm’s parameters were selected. The procedure of finding and optimizing for the XGBoost hyper-parameters was done once the parameters of the PSO algorithm were created. The conventional XGBoost model was developed using the same variables that were applied to regulate the efficiency of the PSO-XGBoost algorithm. The hyper-parameters of the traditional XGBoost model were determined using a grid search approach.

Higher significance is linked with several factors in the PSO-XGBoost model, such as slope, rainfall, fault density, distance from fault, and drainage density, among others. Many researchers describe slope as the representation of the proportion of the vertical distance to the parallel distance between two designated intervals with the tangential angles, and it is considered as an input parameter in susceptibility research. With regard to the steepest drop in slope for elevation, slope is the angle between every surface segment and a horizontal reference point that measures the pace of change in height and facilitates the flow of water (and other materials) in the path of slope. The most prevalent cause of NCL across the world is prolonged and heavy rainfall. Under most circumstances, cultivated land become most vulnerable to natural hazards. An accumulation of precipitation over several days or weeks typically triggers or reactivates deep-seated, slow-moving landslides (e.g., earth flows, slumps), whereas shallow, rapid landslides (debris avalanches, debris flows) on the other hand, generally occur during isolated strong or major storm occurrences. Seismic disturbances may trigger landslides as well. The breach of landslide dams spanning streams during floods can be catastrophic, and flooding may also lead to collapses, thus giving rise to soil erosion and the formation of NCL.

With the widespread advancement in computer science, the ability of predictive models in various fields of earth sciences has also increased. But finding the most appropriate and accurate predictive model for various phenomena, including NCL, is still not easy and requires a lot of effort. In this study, in order to improve the efficiency of the XGBoost model in predicting NCL at the county level, we used the PSO optimization method that is one of the powerful metaheuristic algorithms in optimizing the parameters of machine learning models. Despite, the results showed that the PSO-XGBoost was more efficient than other models, but it is necessary to optimize the parameters of other models with this algorithm to achieve a comprehensive result about hybrid models. On the other hand, there are other metaheuristic methods such as the genetic algorithm that are suggested to be used in future NCL susceptibility modeling studies to evaluate their efficiency.

## Conclusion

Arable land constitutes the essential foundation for ensuring food production, while also serving as the fundamental resource and spatial carrier that safeguards food security, promotes social development, and maintains ecological stability. The phenomenon of non-grain cultivated land (NCL) is a significant factor that restricts the preservation of arable land and the enhancement of food production. Thus, estimation of the NCL susceptibility is a task of prime importance to planners and policy-makers alike. In recent decades, the trend and intensity of NCL in various parts of China has significantly increased. So, from this perspective, the estimation and prediction of NCL susceptibility is a necessary step with regard to sustainable land management practices. In this research, GBM, XGBoost, PSO-GBM and PSO- XGBoost models have been considered for estimating NCL susceptibility. Considering the AUC from ROC and various other statistical indices, the PSO-XGBoost model is considered the most optimal of the models used in this research, and PSO demonstrated a powerful ability to improve the performance of machine learning models. When validation datasets are taken into consideration, the values of sensitivity, specificity, PPV, NPV, and AUC in the PSO-XGB model are 0.93, 0.89, 0.88, 0.93, and 0.96, respectively. Higher significance is linked with several factors in the PSO-XGB model, such as slope, rainfall, fault density, distance from fault, and drainage density, among others. This type of information is helpful to decision-makers and regional planners, when taking the most suitable remedies around sustainable land resource management. The main task for future research is further improvement of the hybridization of predictive models, considering more appropriate parameters in respect to NCL susceptibility.

## Data Availability

The datasets generated and analysed during the current study are not publicly available due [The data are sourced from government classified projects] but are available from the corresponding author on reasonable request.
